# Pulmonary Hypertension in the Course of Interstitial Lung Diseases—A Personalised Approach Is Needed to Identify a Dominant Cause and Provide an Effective Therapy

**DOI:** 10.3390/diagnostics13142354

**Published:** 2023-07-13

**Authors:** Aneta Kacprzak, Witold Tomkowski, Monika Szturmowicz

**Affiliations:** 1st Department of Lung Diseases, National Tuberculosis and Lung Diseases Institute, Plocka 26, 01-138 Warsaw, Poland

**Keywords:** pulmonary hypertension, interstitial lung disease, lung fibrosis, phenotypes, screening, prognosis, treatment, pulmonary arterial hypertension dedicated medications, anti-fibrotic therapy

## Abstract

The prevalence of pulmonary hypertension (PH) complicating interstitial lung diseases (ILDs) is 3.5–15% at an early stage, and up to 90% in ILD patients listed for lung transplantation. In addition, other types of PH may occur in patients with ILDs due to concomitant conditions. Therefore, any significant PH occurring in the setting of ILD requires a proper differential workup. PH increases morbidity and mortality in ILDs. The pathomechanisms underlying PH due to ILD (PH-ILD) are not fully known, and there is no straightforward correlation between the presence or severity of PH-ILD and the severity of ILD. Severe PH in mild ILD without other explanatory causes constitutes a dilemma of differentiating between PH due to ILD and pulmonary arterial hypertension coexisting with ILDs. The heterogeneity and poor prognosis of patients with ILDs coexisting with PH necessitate an individualised approach to the management of this condition. This review presents recent advances in understanding and treatment options in PH-ILD. It also addresses practical issues, such as when to suspect and how to screen for PH in ILD, what are the indications for right heart catheterisation, and how to approach an individual ILD patient to determine the dominant PH cause and apply adequate management.

## 1. Introduction

Pulmonary hypertension (PH) due to lung diseases and/or hypoxia is the second most frequent type of PH [1]. The prevalence of PH in interstitial lung diseases (ILDs) is 3.5–15% at an early stage, 30–50% at the time of advanced disease, and 60–90% in patients listed for lung transplantation [2,3,4,5]. Progressive fibrotic ILDs, especially, constitute a great epidemiological problem [6]. This phenotype appears in 59% of patients with idiopathic pulmonary fibrosis (IPF), 58% of those with fibrotic hypersensitivity pneumonitis (HP), 51% of those with unclassifiable ILDs, and 45% of those with ILD associated with connective tissue disease (CTD-ILD) [6]. Structural lung changes and chronic hypoxia, with subsequent pulmonary vascular remodelling and elevation of pulmonary vascular resistance (PVR), lead to PH development in the course of ILDs [7]. However, it is important to remember that PH in patients with ILD may have different, non-ILD-related causes. PH due to ILD (PH-ILD) is usually mild to moderate. Severe PH in a patient with ILD requires a differential diagnosis that includes other types of PH, such as PH due to heart disease, pulmonary thromboembolic disease, and even pulmonary arterial hypertension (PAH). Determining the predominant cause of PH in ILD and classifying it to the proper PH group is crucial, as this determines its management [1]. PH caused by ILDs belongs to group 3 of the PH clinical classification. It needs to be highlighted that PH associated with sarcoidosis belongs to group 5, regardless of the extent of lung involvement [1].

Prognosis in PH-ILD is poor and worse than in other groups of PH [7,8]. The reported 1-, 3-, and 5-year survival rates are 72–79%, 47–52%, and 37–38% in PH-ILD compared to 85–88%, 72–76%, and 59–66% in PAH [8,9]. The risk of death is two times higher in group 3 PH than in PAH [8]. There are also differences in prognosis within group 3 PH, with ILD patients having a worse prognosis compared to chronic obstructive pulmonary disease (COPD) patients, and IPF patients having a worse prognosis than those with CTD-ILD [8,9,10]. Medications approved for PAH treatment are not recommended for PH-ILD patients due to lack of evidence of their clinical effectiveness [1]. The heterogeneity and poor prognosis of patients with ILD coexisting with PH necessitate an individualised approach to the management of this condition.

The following topics are addressed in the present review: prediction of PH in ILD, indications for right heart catheterisation (RHC), determining the main cause of PH in an individual ILD patient, and personalised treatment approaches.

## 2. Prediction and Screening for PH in ILDs

The symptoms of ILD and PH overlap, with exertional dyspnoea being the most common. Although the symptoms are not specific, they may prompt suspicion of PH complicating ILD in some circumstances; for example, in cases where shortness of breath is out of proportion to the severity of the underlying lung disease, or if symptoms are worsening despite optimal treatment and stable ILD. Predictors of PH in ILDs include: total lung diffusion capacity for carbon monoxide (TLco) < 50% of predicted value, especially with relatively preserved lung volumes [11]; forced vital capacity (FVC) to TLco ratio increased to 1.6–1.8 [4,12]; significant drop in oxygen saturation during a six-minute walking test (6MWT) [12]; increased pulmonary artery (PA) diameter (>29 mm), and/or increased PA diameter to ascending aorta diameter ratio (>0.9), and/or right-to-left ventricle diameter index > 1 on chest CT scan [13,14]; and increased serum levels of brain natriuretic peptide (BNP) or N-terminal BNP pro-peptide (NT-proBNP) [2]. A marked increase in BNP or NT-proBNP serum concentrations in patients with PH-ILD is rare due to slow disease progression; this is usually observed in cases of severe PH with signs of right ventricular failure [15,16,17]. The utility of single parameters and their combinations as clinical predictors of PH are shown in Table 1.

An expert consensus on strategies for PH screening in ILDs has been published recently [20]. The panellists of this multidisciplinary Delphi study identified several triggers for suspicion of PH and recognised echocardiography and NT-proBNP or BNP as useful screening tests [20].

Transthoracic Doppler echocardiography is a non-invasive method for preliminary PH assessment [1]. The peak tricuspid regurgitation velocity (TRV) and additional echocardiographic features allow for estimation of the PH probability level as low, intermediate, or high (Table 2) [1].

Recently, D’Alto et al. validated the above recommendations in a group of patients with various types of PH. They reported that TRV greater than 2.8 m/s was the independent and most important predictor of PH. The value of additional echocardiographic parameters was limited, with inferior vena cava diameter and right atrial area being the least useful [21].

Echocardiography is a good screening tool, but the accuracy of echocardiographic assessment of PH is insufficient to confirm its diagnosis [1]. Respiratory diseases cause additional difficulties in precise TRV measurement. The correlation between pulmonary artery systolic pressure (PASP) calculated from TRV and PASP measured directly during RHC was only moderate (r = 0.609; *p* < 0.01) in lung transplant candidates with ILD or COPD. Echocardiography overestimated or underestimated PASP in 35% and 11.6% of patients, respectively [22]. In another study, 14% of patients with various fibrotic ILDs and echocardiographic high-PH probability did not have PH on RHC, while 40% of patients from the low-probability category showed PH on RHC [23]. Bax et al. proposed a stepwise composite echocardiographic score for the prediction of severe PH (defined as mPAP > 35 mmHg) in ILDs, which works with a sensitivity of 89%, specificity of 71%, positive predictive value of 68%, and negative predictive value of 90% [24].

## 3. RHC in Suspicion of PH Complicating ILDs

Due to the shortcomings of echocardiography, RHC remains the gold standard for PH diagnosis [1]. The recommendations for performing this invasive procedure in patients with suspected group-3 PH have evolved over the years (Table 3). This evolution mainly reflects progress in therapeutic possibilities for the condition. The current European PH guidelines give class I recommendations for RHC in suspicion of PH in patients with lung diseases if the results are expected to affect further management [1].

RHC allows not only for direct measurement of pulmonary artery pressure, but also for more specific characteristics of PH, such as its type and severity. Group 3 PH is a pre-capillary pulmonary hypertension, defined by mean pulmonary artery pressure (mPAP) > 20 mmHg, pulmonary capillary wedge pressure (PCWP) ≤ 15 mmHg, and PVR > 2 Wood units (Wu) [1]. In contrast, post-capillary PH is characterised by PCWP > 15 mmHg and constitutes group 2 of the PH classification [1]. Cardiac diseases and left ventricular failure are common in patients with ILDs [22], and a recent study documented post-capillary PH in 20% of 157 patients diagnosed with ILD and PH [26]. Another advantage of RHC is the possibility of the assessment of PH severity. Severe PH in patients with lung diseases is defined by PVR > 5 Wu [1]. This definition was introduced in the latest European guidelines [1]; the previous definition was based on mPAP and cardiac index [25] (Table 4). The current definition is based on the observation that this PVR threshold is better for prediction of the prognosis in patients with PH associated with respiratory diseases [27]. So defined severe PH-ILD is uncommon and its prevalence is less than 10% among patients with PH-ILD [28].

## 4. Different Phenotypes of PH in ILDs

Most PH-ILD patients have non-severe pulmonary hypertension despite advanced lung disease [7]. Advanced fibrotic ILD presents as reticular opacifications with traction bronchiectasis, and occasionally honeycombing on HRCT [29]. Pulmonary function tests usually show a decrease in lung volumes and TLco, corresponding to the magnitude of HRCT abnormalities. In cases of severe PH associated with ILD, a differential workup should be performed in the search for comorbidities contributing to this state, including left heart disease and venous thromboembolic disease (VTE) [1]. An increased risk for VTE was found in patients with IPF and HP [30,31,32]. However, there is a group of ILD patients with severe PH in whom no additional explanation for high PH can be found. In addition, there is no straightforward correlation between PH presence or severity and the extent of lung fibrosis in the course of ILDs [5]. This suggests the involvement of other factors, not only the reduction of pulmonary vascular bed density, in the development of PH-ILD. The findings from pathologic examinations of explanted lungs revealed vascular remodelling with intimal fibrosis and medial and adventitial hypertrophy in small pulmonary arteries in ILDs, and the remodelling was more pronounced in cases with PH [5,33]. Direct and indirect activation of mediators of apoptosis, angiogenesis, and fibrosis, such as transforming growth factor beta, platelet-derived growth factor, and vascular endothelial growth factor may play a role in vascular disease in ILDs [33]. If vascular remodelling dominates over structural lung disease, a PAH-like or vascular phenotype of PH in a patient with ILD is recognised [1]. Usually, spirometry and lung volumes are better preserved in these cases but impairment of TLco is deeper and hypoxemia is often present. This phenotype of PH in ILD constitutes a major dilemma whether the PH is due to lung disease (group 3 PH) or pulmonary arterial hypertension coexists with ILD (group 1 PH) [7,34]. For these reasons, RHC results should always be interpreted in conjunction with the results of pulmonary function tests and HRCT in patients with ILDs. An aid for distinguishing between vascular and parenchymal phenotypes has been proposed by Nathan et al. (Table 5) [7].

PH in patients with CTDs poses similar challenges, as it may develop via various mechanisms and therefore be assigned to various PH groups—from 1 to 4 [35,36]. PH with predominant vascular disease and non-significant lung fibrosis is classified as PAH. Around 17% of patients classified as PAH-CTD demonstrate mild lung disease [37]. In a cohort of scleroderma patients with lung involvement and PH, 55% had group 3 PH, 24% had group 1 PH, and 21% had group 2 PH [38]. Moreover, various types of PH may coexist in CTD patients and the phenotypes of PH may change in the course of the disease and its treatment [39]. Fayed et al. proposed a diagnostic algorithm for PH in CTDs, based on medical history, echocardiography, HRCT, FVC, and TLco, as well as lung scintigraphy followed by CTPA. The criteria for PH-ILD included the extent of lung fibrosis on HRCT of more than 20% of lung fields, FVC lower than 70% pred., and TLco lower than 30% pred. [40]. The clinical course of CTD-ILD is often benign with non-severe PH developing at the late stage of the disease [41]. Progressive lung fibrosis is seen occasionally, mainly in rheumatoid arthritis with high rheumatoid factor serum levels [42] or in myositis-related syndromes [43].

## 5. Treatment of PH-ILD

It is recommended to optimise treatment of the underlying lung disease in patients with PH-ILD [1,7]. There are two main pharmacological therapies in ILDs—immunosuppressive and anti-fibrotic. Surprisingly, not much data on the effect of immunosuppressive therapy on PH-ILD can be found in the literature. Two retrospective cohort analyses showed that the use of azathioprine or mycophenolate mofetil in combination with low-dose corticosteroids resulted in a significant improvement in TLco in chronic HP; data on PH were not provided [44,45]. An improvement of haemodynamic parameters was achieved with immunosuppressive therapy in PAH associated with systemic lupus erythematosus and mixed connective-tissue disease, but not with scleroderma [46]. Haemodynamic improvement was also shown in PAH-CTD patients treated with a combination of immunosuppressive drugs and PAH-directed therapy [47].

Anti-fibrotic drugs are used to delay disease progression in IPF [48,49] and other fibrotic lung diseases progressing despite immunomodulatory therapy [50,51]. They have not been studied in the context of PH; however, there is increasing evidence that anti-fibrotic therapy does not improve the prognosis in patients with PH due to IPF. Significantly worse survival was observed in IPF patients treated with pirfenidone or nintedanib if they had baseline echocardiographic PASP higher than 50 mmHg [52]. The results from a small prospective study suggest that nintedanib may display different effects in IPF patients depending on the presence or absence of hypoxemia requiring long-term oxygen therapy (LTOT). Patients on LTOT had significantly higher PASP after 48 weeks of nintedanib treatment compared to patients without LTOT, despite both groups having comparable PASP at the baseline [53]. However, a similar increase in PASP was noted in patients with IPF and on LTOT but not on anti-fibrotic treatment in the same study [53]. This would rather indicate a non-drug-related phenomenon. Nonetheless, speculations have appeared that nintedanib may induce inhibition of vascular endothelial growth factor receptor, which, together with chronic hypoxia, results in endothelial cell dysfunction and death [54]. Thus, it may be reasonable to monitor PASP in patients treated with nintedanib, especially in those needing LTOT.

Another important, non-pharmacological intervention in PH-ILD is oxygen supplementation in cases of hypoxemia [1]. There is no direct proof for the beneficial effect of LTOT in patients with PH-ILD, but it is still recommended on the basis of its efficacy in patients with COPD and other forms of PH. A survival benefit and partial reduction in PH progression were shown in COPD patients with respiratory insufficiency receiving LTOT [55,56,57]. Also, LTOT resulted in an improvement in exercise capacity and quality of life in PAH and chronic thromboembolic pulmonary hypertension patients who experienced significant desaturation during 6MWT [58]. Finally, the participants of the Delphi survey agreed that LTOT should be recommended for patients with fibrotic ILDs with severe resting hypoxemia or with a symptomatic exertional decrease in oxygen saturation to less than 89% [59].

## 6. Use of PAH-Directed Medications in PH-ILD

The rationale for the use of PAH-directed therapy in patients with group 3 PH has been discussed for many years. Especially, patients with the vascular phenotype of PH-ILD seem to be a tempting target. Although positive results were reported for individual patients and a registry cohort [16], the results from the majority of randomised clinical trials (RCTs), conducted in the period 2010–2021 and enrolling patients with IPF and other fibrotic ILDs, were negative (Table 6) [60,61,62,63]. Two studies, one with riociguat (RISE IIP study) and the other with ambrisentan (ARTEMIS-IPF study), had to be terminated early because of unfavourable outcomes observed in patients treated with active drugs [61,62]. Post hoc analysis of the RISE IIP study showed that the increased death rate in patients treated with riociguat was associated with low baseline TLco [64].

On the other hand, there have been few RCTs showing benefits of PAH medications in PH-ILD. The subgroup analysis of the STEP-IPF study revealed sildenafil efficacy in IPF patients with right ventricle dysfunction (RVD) on echocardiography [64]. Recently, reports from two RCTs with inhaled PAH agents—nitric oxide (NO) and treprostinil—have been published [66,67]. The INCREASE study with inhaled treprostinil showed a significant improvement in 6MWT distance and NT-proBNP levels, as well as a decrease in the clinical worsening rate in the arm of active treatment in patients with PH-ILDs [67]. Following these results, the latest European PH guidelines recommend consideration of inhaled treprostinil in patients with PH-ILDs irrespective of PH severity, while the use of riociguat and ambrisentan is not recommended [1]. There is still no consensus on the use of phosphodiesterase 5 inhibitors (PDE5is) due to the lack of evidence of their efficacy and safety in PH-ILDs. Therefore, only conditional and based on very low-quality evidence recommendations have been introduced, according to which PDE5is may be considered in patients with severe PH-ILD, but are not recommended in patients with non-severe PH-ILD [1]. The discussion on the use of PDE5is in patients with PH-ILDs is still ongoing, though. The survival benefit of PDE5is (sildenafil and tadalafil) therapy in patients with PH due to various ILDs has recently been shown in a large retrospective study [68]. A total of 89% of the included patients had severe PH according to the 2018 definition [7], and 66% of them presented with echocardiographic signs of RVD. The largest benefit of the PDE5is was noted in patients without RVD [68]. Moreover, the beneficial effect of sildenafil on survival in IPF patients was also shown in a recent meta-analysis [69]. Future studies are urgently needed to investigate the role of PDE5is in PH-ILDs. Another question that is still open is the timing of PAH-targeted therapy commencement in patients with PH-ILDs. A multi-institutional prospective cohort study showed better survival in patients with the PAH-like phenotype of PH associated with respiratory diseases if they started PAH treatment within 2 months from RHC, as compared to those in whom the treatment was delayed [70]. The survival prognosis was significantly better in responders to PAH therapy than in non-responders. The response to therapy was defined as either an improvement in the World Health Organisation functional class, a decrease in PVR > 15%, or an increase in 6MWT distance > 15% at the first follow-up visit after a mean of 332 days [70].

One of the major concerns associated with the use of pulmonary vasodilators in group 3 PH is a worsening of gas exchange due to an increase in the pulmonary shunt [63,71]. This complication may result from redirecting the blood flow to the fibrotic areas of the lungs [71]. It has been noted that the impact on ventilation/perfusion mismatch in PH-ILDs depends on a class of PAH-targeted medications. Sildenafil, in contrast to epoprostenol, did not affect the pulmonary shunt and even improved the arterial partial pressure of oxygen [71]. No deterioration of oxygen saturation in end-stage IPF patients treated with sildenafil was observed in the STEP-IPF trial, either [60]. In an ex vivo/in vitro study, the pulmonary vaso-relaxant effect of sildenafil depended on a degree of vascular remodelling; it was significantly more marked in lungs affected with IPF without PH than in IPF-PH [72]. This phenomenon may be caused by an insufficient amount of endogenous NO in the areas of remodelled pulmonary arteries.

No deterioration in arterial blood oxygenation was noted in trials using inhaled vasoactive drugs, most probably due to their selective penetration to better ventilated parts of the lungs [66,67].

## 7. Combination of Anti-Fibrotic and PAH-Directed Therapies in PH-ILD

Combination therapy with PAH-targeted medications and anti-fibrotic medications has been tested in two RCTs, one with sildenafil as an add-on therapy to pirfenidone [73] and the second with sildenafil added to nintedanib (INSTAGE study) [74]. The former study, with sildenafil and pirfenidone, was conducted in a cohort of patients with advanced IPF and concomitant PH diagnosed with either echocardiography or RHC. This study failed to demonstrate the benefit from sildenafil addition to pirfenidone in respect of disease progression [73]. A population enrolled into the INSTAGE trial consisted of patients with IPF and low TLco (≤35% predicted). The study assessed a change in health-related quality of life as a primary end-point. No superiority of combination therapy with nintedanib and sildenafil was observed compared to nintedanib alone [74]. Additional analyses showed a significant preventive effect of sildenafil plus nintedanib on the BNP level increase compared to nintedanib alone in the subgroup of patients who presented with echocardiographic signs of right heart dysfunction at the baseline [75]. In the subgroup of patients without right heart dysfunction, no significant increase in BNP levels was present in any of the treatment arms [75].

## 8. Future Directions in the Treatment of PH-ILD

New properties of PAH-targeted medications have recently been discovered and investigated. Treprostinil has shown its anti-fibrotic potency through the activation of prostaglandin E receptor 2, prostaglandin D receptor 1, and peroxisome proliferator-activated receptors. An inhibition effect on cell proliferation and collagen synthesis has been observed in vitro [76]. Also, a post hoc analysis of the INCREASE study revealed a significant improvement in FVC in patients treated with inhaled treprostinil compared to a placebo [77], as well as a reduction in multiple disease progression events, such as a decline in 6MWT distance, exacerbation of the underlying lung disease, FVC decline, cardiopulmonary hospitalizations, lung transplantation, and death [78]. The TETON study has been started to investigate the efficacy and safety of inhaled treprostinil in IPF, with the absolute change in FVC at week 52 set as a primary endpoint [79]. Also, trials focusing on new anti-fibrotic drugs that act through phosphodiesterase inhibition and their possible impact on vascular remodelling in IPF have been started recently [80,81].

In recent years, there has been increasing interest in the percutaneous transcatheter treatment of tricuspid valve regurgitation. The procedure has been found to be safe and effective in improving the symptoms of right heart failure and quality of life in patients with symptomatic, moderate or greater tricuspid regurgitation in the course of various conditions [82,83]. Patients with significant pre-capillary PH have not been included in studies exploring this therapeutical option, so its safety and clinical benefit in this set of patients remains unknown.

The clinical significance of PH-ILD, the importance and desired directions of future research on its pathomechanisms and therapeutic options, have recently been highlighted by the Innovative Drug Development Initiative of the Pulmonary Vascular Research Institute [84].

## 9. Summary

Early recognition of PH in the course of ILD is important as it may have therapeutic and prognostic consequences. Differential diagnosis of PH in ILD has to include other causes of PH, such as left heart disease and VTE. The predominant cause of PH may change in the course of ILD; thus, therapy modifications have to be considered on an individual basis.

In patients with PH due to advanced lung disease, optimal therapy of the underlying lung disease and LTOT, if indicated, are recommended; however, their impact on PH and survival remains uncertain.

Patients with severe PH due to lung fibrosis have a poor prognosis despite anti-fibrotic therapy.

RHC should be performed in patients who are considered for any PAH-directed therapy to confirm precapillary PH and assess its severity. Inhaled treprostinil may be considered for PH-ILD to improve exercise capacity. PDE5is may be considered in patients with severe PH-ILD.

RCTs with PDE5is are urgently needed to investigate the potential benefit of such treatment in patients with PH-ILD confirmed on RHC. RCTs with combination therapy consisting of PDE5is or treprostinil and anti-fibrotic medications are needed to assess the therapeutic potential in patients with PH-ILD confirmed on RHC.

## Figures and Tables

**Table 1:** Prediction of PH in ILDs based on single parameters and their combinations.

Author	No. of Patients, Disease	Modality and Criteria Used for PH Diagnosis	Predictors	Model of Prediction
Furukawa et al. [11]	273, IPF	RHC mPAP ≥ 21 mmHg	1 point for each criterion: TLco < 50%, CTPA PA/Ao ≥ 0.9, PaO_2_ < 80 mmHg	Total scoring sensitivity for PH on RHC: 3 points–65.4%, 0 points–6.7%
Sonti et al. [18]	105, IPF	RHC mPAP ≥ 25 mmHg	RVSP, FVC/TLco, PA/Ao	mPAP = (−14) + 20.3 × (PA/Ao) + 2.6 × (FVC/TLco) + 0.3 × RVSP sensitivity 80%, specificity 68%
Dybowska et al. [19]	70, HP	TTE RVSP > 36 mmHg	TLco < 42% 6MWT desaturation > 8%	Sensitivity 62%, specificity 89%; sensitivity 58%, specificity 77%
Sobiecka et al. [12]	93, various ILDs	TTE PH possible or likely according to European PH guidelines [1]	3 points for each criterion: Age > 53 years, 6MWT distance < 507.5 m 2 points for each criterion: SpO_2_ < 93%, TLC/TLco > 1.67	Total scoring >6 points: sensitivity 64%, specificity 94%

IPF—idiopathic pulmonary fibrosis, RHC—right heart catheterisation, mPAP—mean pulmonary artery pressure, TTE—transthoracic echocardiography, RVSP—right ventricle systolic pressure, TLco—total lung diffusion capacity for carbon monoxide, CTPA—computed tomography pulmonary angiogram, PA—pulmonary artery, Ao—aorta, PaO_2_—partial pressure of oxygen, PH—pulmonary hypertension, FVC—forced vital capacity, HP—hypersensitivity pneumonitis, 6MWT—6-min walking test, ILDs—interstitial lung diseases, SpO_2_—oxygen saturation, TLC—total lung capacity.

**Table 2:** Doppler echocardiography for the assessment of PH probability [1].

TRV Max	Additional Echo Signs of PH	Echocardiographic Probability of PH
≤2.8 m/s	absent	low
≤2.8 m/s	present	intermediate
2.9–3.4 m/s	absent	intermediate
2.9–3.4 m/s	present	high
>3.4 m/s	absent or present	high

TRV—tricuspid regurgitation velocity, PH—pulmonary hypertension.

**Table 3:** Recommendations for RHC in group 3 PH according to European guidelines and expert recommendations [1,7,25].

European PH Guidelines 2015 [25]	Expert Recommendations 2018 [7]	European PH Guidelines 2022 [1]
RHC is not recommended for suspected PH in patients with lung disease, unless therapeutic consequences are to be expected (e.g., lung transplantation, alternative diagnoses such as PAH or CTEPH, potential enrolment in a clinical trial)—class of recommendation III, level of evidence C.	RHC should be performed in patients with chronic lung disease when significant PH is suspected and the patient’s management will likely be influenced by the RHC results, including referral for transplantation, inclusion in clinical trials or registries, treatment of unmasked left heart dysfunction, or compassionate use of therapy.	In patients with lung disease and suspected PH, RHC is recommended if the results are expected to aid management decisions (assessment for surgical treatment, suspicion of PAH or CTEPH, when further information will aid phenotyping of disease and consideration of therapeutic interventions)—class of recommendation III, level of evidence C.

RHC—right heart catheterisation, PH—pulmonary hypertension, PAH—pulmonary arterial hypertension, CTEPH—chronic thromboembolic pulmonary hypertension.

**Table 4:** Changing definition of severe PH-ILD.

European PH Guidelines 2015 [25]	2018 Expert Recommendations [7]	European PH Guidelines 2022 [1]
mPAP > 35 mmHg or mPAP ≥ 25 mmHg in the presence of a low cardiac index (CI < 2.5 L/min, not explained by other cause)	mPAP ≥ 35 mmHg or mPAP ≥ 25 mmHg with low cardiac index < 2.0 L/min	mPAP > 20 mmHg PVR > 5 Wood units

PH—pulmonary hypertension, ILD -interstitial lung disease, mPAP—mean pulmonary artery pressure, CI—cardiac index, PVR—pulmonary vascular resistance.

**Table 5:** Principles of differential diagnosis between group 1 PH (PAH) with coexisting ILD and group 3 PH (due to lung disease) according to Nathan et al. (modified) [7].

Criteria Favouring PAH—Group 1	Testing	Criteria Favouring PH-ILD—Group 3
*Extent of lung disease*		
Normal or mildly impaired FVC > 70% pred. (IPF) TLco in relation to restrictive changes	**Pulmonary function testing**	Moderate to severely impaired FVC < 70% pred. (IPF) TL.co corresponds to restrictive changes
Absence or only modest parenchymal changes	**HRCT**	Characteristic parenchymal changes
*Haemodynamic profile*		
Moderate to severe PH	**RHC** **TTE**	Mild to moderate PH
*Ancillary testing*		
Present	**Further PAH risk factors (HIV, CTD, BMPR2 mutation)**	Absent

PAH—pulmonary arterial hypertension, PH—pulmonary hypertension, PH-ILD—pulmonary hypertension associated with interstitial lung disease, FVC—forced vital capacity, IPF—idiopathic pulmonary fibrosis, TLco—total lung diffusion capacity for carbon monoxide, HRCT—high-resolution computed tomography, RHC—right heart catheterisation, TTE—transthoracic echocardiography, HIV—human immunodeficiency virus, CTD—connective tissue disease, BMPR2—bone morphogenic protein receptor type 2.

**Table 6:** The results of RCTs on the efficacy of pulmonary vasodilators in patients with PH-ILDs.

Source	Year	RCT Acronym	Study Population	Drug	Duration	Primary End-Point	Result
Zisman et al. [60]	2010	STEP-IPF	IPF 180 pts	Sildenafil	12 weeks	Change in 6MWT	N
Han et al. [65]	2013	STEP-IPF substudy	IPF/with TTE data 119 pts	Sildenafil	12 weeks	Change in 6MWT	P
Raghu et al. [62]	2013	ARTEMIS-IPF	IPF 19 pts	Ambrisentan	12 months	Combined	N
Corte et al. [63]	2014		IPF/fNSIP 60 pts	Bosentan	16 weeks	Change in PVR	N
Nathan et al. [61]	2019	RISE-IIP	IIP 147 pts	Riociguat	12 months	Change in 6MWT	N
Nathan et al. [66]	2020		PF-ILD 41 pts	Inhaled NO	8 weeks	Change in actigraphy	P
Waxman et al. [67]	2021	INCREASE	IIP 326 pts	Inhaled treprostinil	16 weeks	Change in 6MWT	P

RCT—randomised clinical trial, pts—patients, IPF—idiopathic pulmonary fibrosis, 6MWT—6-min walking test, TTE—transthoracic echocardiography, fNSIP—fibrotic non-specific interstitial pneumonitis, PVR—pulmonary vascular resistance, IIP—idiopathic interstitial pneumonitis, PF-ILD—progressive fibrosing interstitial lung disease, NO—nitric oxide, N—negative, P—positive.

## Data Availability

Not applicable.
